# Acute Appendicitis Before, During, and After the Initial COVID-19 Pandemic Lockdown: A Single-Center Retrospective Review in Arizona

**DOI:** 10.7759/cureus.105426

**Published:** 2026-03-18

**Authors:** Zola Trotter, Colin Hurkett, Katherine Barlow, Eric Jackson, Tamlyn Tarzia, Bikash Bhattarai

**Affiliations:** 1 Emergency Medicine, Valleywise Health Medical Center, Phoenix, USA; 2 Psychiatry, University of Washington, Seattle, USA; 3 Critical Care, Valleywise Health Medical Center, Phoenix, USA; 4 Biostatistics, Valleywise Health Medical Center, Phoenix, USA

**Keywords:** complicated appendicitis, covid-19, hospital length of stay, non-operative management, simple appendicitis

## Abstract

Introduction: The COVID-19 pandemic introduced novel challenges for healthcare systems. This study compares the incidence of simple versus complicated appendicitis, hospital length of stay (LOS), and non-operative management (NOM) during the initial five-month COVID-19 lockdown of 2020 against various pre- and post-reference periods.

Methods: We conducted a retrospective analysis of all patients diagnosed with appendicitis between March 31, 2017, and August 31, 2022. Data from the reference period (March 31-August 31, 2020) were compared with shorter identical-calendar-period comparators from 2017 to 2022, as well as with longer comparator time periods consisting of consecutive 36 months preceding and 24 months succeeding the reference period.

Results: A total of 1094 patients were included in the analysis, of which 87 were in the reference period. The shorter comparator periods included 63, 74, 92, 106, and 109 patients in 2017, 2018, 2019, 2021, and 2022, respectively. The longer comparator periods comprised 540 patients in the 36 months preceding the reference period and 467 patients in the 24 months succeeding it. Mean age and gender distribution were similar across all comparator periods, except in post-reference periods, where mean heart rates decreased and CT scan use increased. There were no significant differences in the rates of complicated appendicitis between the reference period (26.5%) and shorter comparator periods (34.9% in 2017, 17.6% in 2018, 19.6% in 2019, 33.0% in 2021, and 23.9% in 2022; p = 0.07). Similarly, complicated appendicitis rates were unchanged when compared to the longer comparator periods (24.1% preceding vs. 25.9% succeeding; p = 0.76). LOS adjusted for clinical and demographic features was significantly longer in 2018 (p = 0.03), 2019 (p = 0.01), and 2022 (p = 0.01) when compared to the reference period and to 2021. NOM did not differ significantly across the shorter comparator periods (p = 0.51) but was higher in the 24 months succeeding (17.3%) than the 36 months preceding (9.1%; p < 0.001).

Conclusions: During the initial COVID-19 lockdown reference period, the proportion of simple versus complicated appendicitis remained unchanged. Hospital LOS was shorter during the reference period and in 2021 compared to other periods. Although unchanged during the reference period, NOM rates increased over the succeeding years.

## Introduction

Acute appendicitis is the most common surgical emergency, with a lifetime risk of 6.7% for females and 8.6% for males in the United States, and a peak incidence between 10 and 20 years of age [1]. The commonly accepted pathophysiology is that simple appendicitis develops from luminal obstruction, often by fecalith or lymphoid hyperplasia. This is followed by progression involving appendiceal distension and inflammation, which eventually leads to complicated appendicitis (perforation, abscess, or gangrene) [2]. Chronic appendicitis is a distinct, long-term inflammatory process that may manifest as persistent or episodic symptoms over months or years and was categorized as an exclusion criterion in this study. The conventional rationale holds that the advancement from simple to complicated appendicitis is time-sensitive, and thus, early presentation is critical to preventing morbidity.

The coronavirus-2019 (COVID-19) pandemic significantly altered healthcare systems globally. Measures implemented to mitigate the disease burden included government-mandated stay-at-home orders and the restructuring of hospital policies, such as the postponement of non-emergent surgeries. The state of Arizona enacted stay-at-home (lockdown) orders on March 31, 2020 [3].

Previous studies have characterized a significant decline in emergency department (ED) utilization alongside a concurrent rise in the delayed presentation of acute medical and surgical conditions during the systemic acute viral syndrome (SARS) viral epidemic [4] and the COVID-19 pandemic [5]. In recent years, investigators across continents have published articles addressing appendicitis during the COVID-19 pandemic, many of which found a higher frequency of complicated appendicitis [6]. It is important to investigate such findings in different regions due to variations in public health responses and demographics.

Our primary objective was to compare the incidence of simple and complicated appendicitis during the initial lockdown period (first five months of the stay-at-home orders from March 31 to August 31), with identical calendar time frames from preceding and succeeding years. We also compared this initial lockdown phase to the continuous 36 months preceding and the 24 months succeeding. Secondary objectives were to investigate the mean hospital length of stay (LOS) and the use of non-operative management (NOM) during these time frames. We hypothesized that during the initial COVID-19 lockdown period, there would be increased proportions of complicated appendicitis, shorter LOS, and greater use of NOM.

## Materials and methods

Study design

We performed a single-center, retrospective analysis of patients aged 0-99 years diagnosed with appendicitis before, during, and after the early phase of the COVID-19 pandemic (March 31-August 31, 2020). The study period extended from March 31, 2017, to August 31, 2022. Our institution, Valleywise Medical Health Center, is a public community teaching hospital located in an urban setting. 

Data collection and definitions

We queried electronic charts by identifying relevant ICD-10 codes, which included diagnoses for acute appendicitis, complicated appendicitis, acute appendicitis with abscess, peritonitis, perforation, gangrene, phlegmon, and unspecified appendicitis. A manual, individualized chart review of electronic medical records was subsequently performed to verify all information. We collected data on demographics, vital signs, white blood cell count (WBC), radiology results, management strategies, and hospital LOS in days. Simple appendicitis was defined as appendicitis without evidence of perforation, gangrene, abscess, or phlegmon on ultrasound (US) or computed tomography (CT) scans, as reported by an attending radiologist. Complicated appendicitis was defined as appendicitis with radiographic evidence of perforation, gangrene, abscess, or phlegmon.

To limit confounding factors, we excluded patients who (1) had chronic appendicitis, (2) had return visits for postoperative complications of acute appendicitis, (3) had immunocompromised states (e.g., malignancies or active chemotherapy), or (4) had been transferred from or to another facility for the management of acute appendicitis.

To account for potential variance in appendicitis due to seasonality [7] and associated environmental and viral exposures [8], we compared the reference period (the five months of the initial COVID-19 lockdown: March 31-August 31, 2020) to five comparator groups with identical calendar time frames in 2017, 2018, 2019, 2021, and 2022, as well as to two comparator groups comprising the entire 36 months preceding and the entire 24 months succeeding the reference period.

Statistical analysis

The calendar period of March 31-August 31, 2020, was used as the reference group for both short identical and long continuous time frames across all statistical models. A p-value < 0.05 was considered statistically significant. All statistical analyses were performed using SAS EG 7.13 (SAS Inc., Cary, NC, USA). Patient age was categorized into five separate strata: 0-10, 11-18, 19-40, 41-60, and >60 years. We reported the means and standard deviations for continuous variables and frequencies and proportions for categorical variables. Patient demographics were stratified by time categories compared across the varied time periods using chi-square tests for categorical variables and the Wilcoxon rank-sum test for continuous variables. The Cochran-Armitage test for trend was used to evaluate annual trends. We evaluated the LOS in days for each time category by fitting a mixed-effects regression model adjusted for ethnicity, gender, age, and type of appendicitis (simple versus complicated). The month of diagnosis was included as a random effect to account for potential temporal variability across months; however, the month itself was not the primary variable of interest. We estimated the odds of receiving NOM for each time category across our full cohort by employing logistic regression models adjusted for patient demographics and appendicitis type.

Ethics statement

This study was approved by the institutional review board at our institution (IRB no. 2020-063). Due to the retrospective nature of the study, informed consent was waived. All authors received standard education on research ethics and data collection protocols.

## Results

A total of 1,152 patient charts were reviewed. Fifty-eight patients were excluded based on predefined criteria, yielding a final cohort of 1,094 patients spanning the study period from March 31, 2017, to August 31, 2022. 

Table 1 outlines the patient population characteristics, which were largely homogeneous across the time categories. Notable exceptions during the post-reference periods (both the shorter 2021 and 2022 time frames and the consecutive 24 months following the reference period) were significantly lower mean heart rates and an increased use of CT scans as the definitive diagnostic modality.

**Table 1:** Characteristics of the patient population The reference period is from March 31 to August 31, 2020. Shorter time frames include five identical calendar periods from March 31 to August 31 for the years 2017, 2018, 2019, 2021, and 2022. Longer time frames include two different time frames: the pre-COVID-19 lockdown (from March 31, 2017, to March 31, 2020) and the post-COVID-19 lockdown (from August 31, 2020, to August 31, 2022). Diagnostic modalities, including CT, MRI, and US, were mutually exclusive. We reported on the specific imaging study that established the final diagnosis. Although some patients underwent multiple examinations, we report only the definitive modality used for diagnostic confirmation. Individual year MRI counts reflect only the March 31-August 31 time frame. Aggregated totals include procedures performed throughout the entire calendar year, accounting for the non-zero values observed. The statistical methods used were chi-square tests for categorical variables and Wilcoxon rank-sum test for continuous variables. The summed figures of the individual years do not add up to the total N for 36 months prior (n = 540) and 24 months prior (n = 467), because yearly data in the individual years is restricted to the March 31-August 31 period for each respective year, excluding full calendar year totals. ^α^Comparison of the reference time to the five short-term time categories; represents a comparison against a pooled five-year historical average to stabilize variance. ^β^Comparison of the reference time lockdown to two longer time frames; compares multi-year aggregates using rate-standardization to account for unequal period lengths. ^*^Statistically significant result for alpha level < 0.05. --Negligible values or p-values not listed for zero. bpm: breaths per minute, WBC: white blood cell, CT: computed tomography, MRI: magnetic resonance imaging, US: ultrasound.

	Reference period	Shorter time frames						Longer time frames		
	2020 (n = 87)	2017 (n = 63)	2018 (n = 74)	2019 (n = 92)	2021 (n = 106)	2022 (n = 109)	p-value^α^	36 months prior (n = 540)	24 months after (n = 467)	p-value^β^
Age (years), mean ± SD	28.8 ± 17.2	22.2 ± 18.3	23.6 ± 14.1	27.5 ± 18.4	27.3 ± 14.6	30.6 ± 15.1	<0.001^*^	25.1 ± 16.9	29.9 ± 15.6	<0.001^*^
Age, n (%)										
0-10	11 (12.6)	19 (30.2)	14 (18.9)	20 (21.7)	11 (10.4)	8 (7.3)	<0.001^*^	118 (21.9)	48 (10.3)	0.04^*^
11-18	18 (20.7)	20 (31.8)	18 (24.3)	19 (20.7)	22 (20.8)	15 (13.8)		130 (24.1)	84 (18)	
19-40	38 (43.7)	12 (19.1)	31 (41.9)	28 (30.4)	52 (49.1)	58 (53.2)		190 (35.2)	210 (45)	
41-60	13 (14.9)	7 (11.1)	10 (13.5)	22 (23.9)	19 (17.9)	24 (22)		83 (15.4)	111 (23.8)	
>60	7 (8)	5 (7.9)	1 (1.4)	3 (3.3)	2 (1.9)	4 (3.7)		19 (3.5)	14 (3)	
Male, n (%)	50 (57.5)	39 (61.9)	44 (59.5)	56 (60.9)	57 (53.8)	69 (63.3)	0.78	312 (57.8)	263 (56.3)	0.9
Hispanic, n (%)	74 (85.1)	55 (87.3)	71 (96)	83 (90.2)	94 (88.7)	94 (86.2)	0.30	470 (87)	396 (84.8)	0.58
Vital signs										
Temperature (°C), mean ± SD	37 ± 0.7	37.3 ± 0.9	36.9 ± 0.7	36.9 ± 0.8	36.8 ± 0.4	36.7 ± 0.5	<0.001^*^	36.9 ± 0.8	36.7 ± 0.4	<0.001^*^
Heart rate (bpm), mean ± SD	100 ±25	100 ±27	96 ±25	94 ± 25	82 ± 16	80 ±15	<0.001^*^	96 ± 23.9	82.4 ± 15.9	<0.001^*^
Respiratory rate (bpm), mean ± SD	19 ± 4	19 ± 3	18 ± 3	19 ± 3	19 ± 3	18 ±3	<0.001^*^	19 ± 3	18 ± 3.4	<0.001^*^
WBC (x10^9^/L), mean ± SD	15.2 ± 4.3	15.6 ± 5	16 ± 4.6	15.3 ± 4.8	14.1 ± 2.7	15.1 ± 4.4	0 .01^*^	15.4 ± 4.8	14.2 ± 3.1	<0.001^*^
Diagnostic imaging, n (%)										
CT	73 (83.9)	48 (76.2)	59 (79.7)	71 (77.2)	96 (90.6)	105 (96.3)	<0.001^*^	437 (80.9)	433 (92.7)	<0.001^*^
MRI	0	0	0	0	0	0	--	1 (0.2)	0	--
US	14 (16.1)	14 (22.2)	14 (18.9)	21 (22.8)	10 (9.4)	4 (3.7)	<0.001^*^	100 (18.5)	34 (7.3)	<0.001^*^
No imaging	0	1 (1.6)	1 (1.4)	0	0	0	--	2 (0.4)	0	--

**Figure 1:**
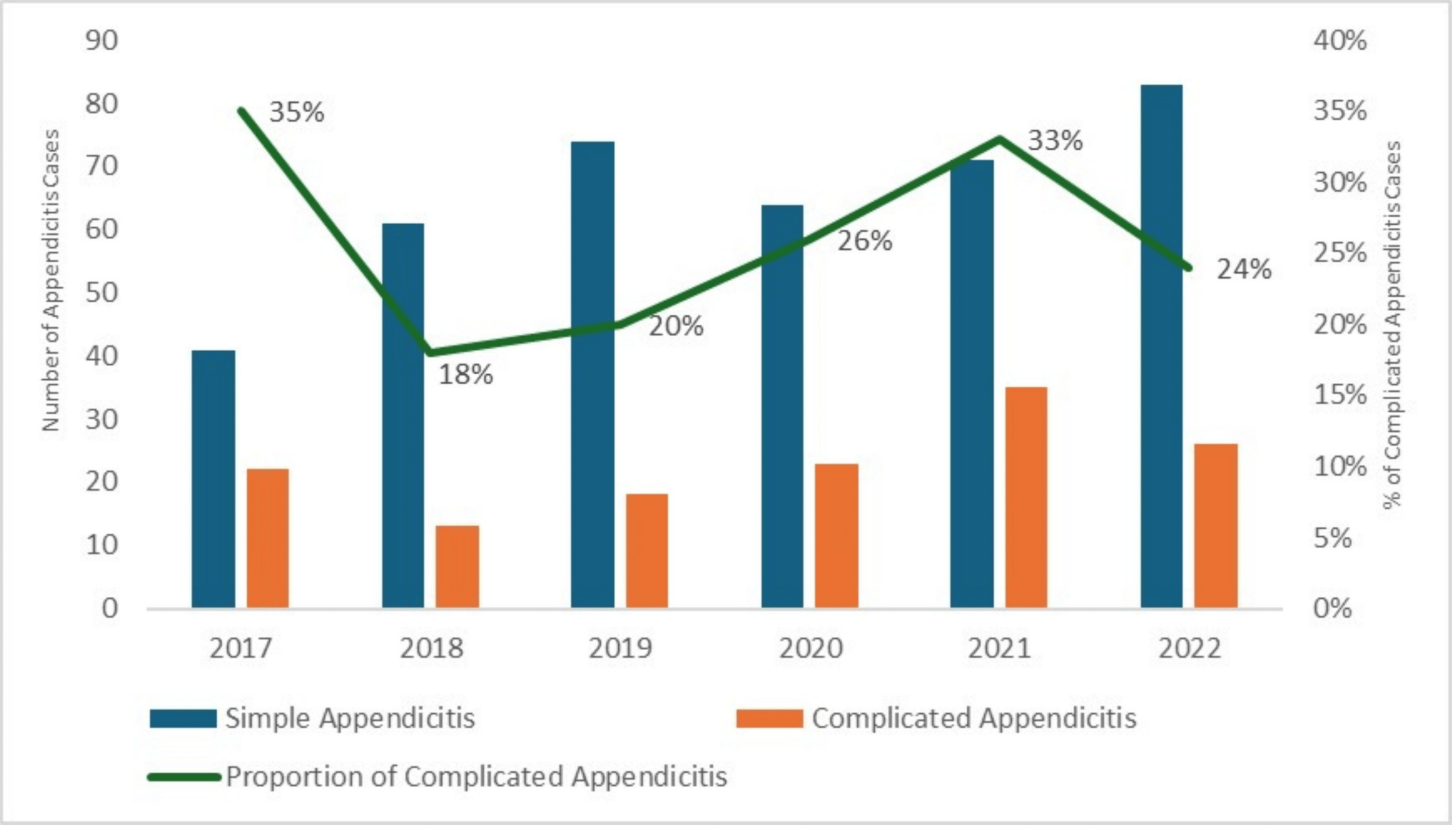
Proportional variation of simple and complicated appendicitis across shorter time frames Shorter time frames include five identical calendar periods from March 31 to August 31 for the years 2017, 2018, 2019, 2021, and 2022.

Figure 1 illustrates the trends in the proportions of simple appendicitis, complicated appendicitis, and their proportional variation across the shorter time frames. When comparing other time frames to the initial COVID-19 pandemic lockdown period in 2020, the proportion of complicated appendicitis cases fluctuated across the years, without demonstrating a clear upward or downward trend (p = 0.07).

Table 2 presents the primary outcomes, total ED visits, and secondary outcomes for each time frame. We observed a nadir in total ED visits during the initial COVID-19 lockdown phase, followed by a return to pre-pandemic numbers in the ensuing two years. Simple appendicitis cases were considerably lower in the 2017 time frame compared to other periods; this occurred without a significant change in ED visits or frequency of complicated appendicitis.

**Table 2:** Primary and secondary outcomes The reference period is from March 31 to August 31, 2020. Shorter time frames include five identical calendar periods from March 31 to August 31 for the years 2017, 2018, 2019, 2021, and 2022. Longer time frames include two different time frames: the pre-COVID-19 lockdown (from March 31, 2017, to March 31, 2020) and the post-COVID-19 lockdown (from August 31, 2020, to August 31, 2022). Statistical methods used were Cochran-Armitage test to evaluate annual trends; chi-square tests for categorical variables; and Wilcoxon rank-sum test for length of stay. Total emergency department (ED) visits were included to draw a reference to total volumes during each time frames. ^α^Comparison of the reference time to the five short-term time categories; represents a comparison against a pooled five-year historical average to stabilize variance. ^β^Comparison of the reference time lockdown to two longer time frames; compares multi-year aggregates using rate-standardization to account for unequal period lengths. ^*^Statistically significant result for alpha level < 0.05. "ref" served as the reference category for statistical comparisons; therefore, no p-value is reported for this group. "n/a" represents the p-values not listed and not applicable.

	Reference period	Shorter time frames						Longer time frames		
	2020 (n = 87)	2017 (n = 63)	2018 (n = 74)	2019 (n = 92)	2021 (n = 106)	2022 (n = 109)	p-value^α^	36 months prior (n = 540)	24 months after (n = 467)	p-value^β^
Primary outcome										
Simple appendicitis, n (%)	64 (73.6)	41 (65.1)	61 (82.4)	74 (80.4)	71 (67)	83 (76.1)	ref	410 (75.9)	346 (74.1)	ref
Complicated appendicitis, n (%)	23 (26.4)	22 (34.9)	13 (17.6)	18 (19.6)	35 (33)	26 (23.9)	.07	130 (24.1)	121 (25.9)	.76
Total ED visits, n	16,167	28, 472	27, 325	27, 479	23,086	25,851	n/a	201,795	105,388	n/a
Secondary outcomes										
Non-operative management	8 (9.2)	6 (9.5)	4 (5.4)	6 (6.5)	12 (11.3)	14 (12.8)	ref	49 (9.1)	81 (17.3)	ref
Operative management	79 (90.8)	57 (90.5)	70 (94.6)	86 (93.5)	94 (88.7)	95 (87.2)	0.51	491 (90.9)	386 (82.7)	<0.001^*^
Hospital length of stay (days), mean ± SD	1.6 ± 1.4	1.9 ± 1.4	1.8 ± 1.5	1.9 ± 1.6	1.86 ± 1.6	1.9 ± 1.9	0.06	1.8 ± 1.8	1.7 ± 1.6	0.08

Regression-based estimates for LOS are detailed in Table 3 and Table 4. The overall LOS was shortest during the initial 2020 COVID-19 lockdown and the 2021 time frame. Significantly longer LOS were observed in 2018 (p = 0.03), 2019 (p = 0.01), and 2022 (p = 0.01). Complicated appendicitis was associated with significantly longer LOS (p < 0.001). No significant difference in LOS was noted between gender and ethnicity. LOS was shorter for age groups 11-18 (p = 0.01) and 41-60 (p = 0.01), relative to the reference group of patients over 60 years old.

**Table 3:** Mixed-effects regression models comparing length of stay across shorter time frames The reference period is from March 31 to August 31, 2020. Shorter time frames include five identical calendar periods from March 31 to August 31 for the years 2017, 2018, 2019, 2021, and 2022. ^β^Linear mixed-effects model for length of stay using a random intercept for month, robust standard errors, as well as age, gender, ethnicity, and appendicitis type as factors and covariates. ^*^Statistically significant result for alpha level < 0.05. "ref" served as the reference category for statistical comparisons; therefore, no p-value is reported for this group.

Time period	Unadjusted beta-coefficient (95% CI)	p-value	Adjusted beta-coefficient (95% CI)^β^	p-value
Intercept	1.54 (1.19, 1.89)	<0.001^*^	1.48 (0.87, 2.09)	0.01^*^
2020 (ref)	ref	ref	ref	ref
2017	0.40 (-0.17, 0.97)	0.17	0.19 (-0.01, 0.39)	0.07
2018	0.27 (-0.20, 0.74)	0.26	0.45 (0.04, 0.86)	0.03^*^
2019	0.38 (0.11, 0.65)	0.01^*^	0.54 (0.17, 0.91)	0.01^*^
2021	0.33 (-0.20, 0.86)	0.23	0.23 (-0.18, 0.64)	0.26
2022	0.34 (-0.05, 0.73)	0.1	0.44 (0.09, 0.79)	0.01^*^
Diagnosis type				
Simple appendicitis	1.25 (1.23, 1.27)	ref	ref	ref
Complicated appendicitis	2.22 (1.79, 2.65)	<0.001^*^	2.21 (1.80, 2.62)	<0.001^*^
Gender				
Male	1.90 (1.70, 2.10)	ref	ref	ref
Female	-0.20 (-0.40, -0.00)	0.05	-0.04 (-0.18, 0.10)	0.56
Ethnicity				
Non-Hispanic	1.97 (1.54, 2.40)	ref	ref	ref
Hispanic	-0.17 (-0.68, 0.34)	0.55	0.03 (-0.46, 0.52)	0.9
Age category				
0-10	-0.43 (-1.14, 0.28)	0.23	-0.16 (-0.98, 0.66)	0.7
11-18	-1.27 (-1.78, -0.76)	<0.001^*^	-0.85 (-1.46, -0.24)	<0.001^*^
19-40	-0.98 (-1.55, -0.41)	<0.001^*^	-0.54 (-1.19, 0.11)	<0.001^*^
41-60	-1.18 (-1.73, -0.63)	<0.001^*^	-0.72 (-1.29, -0.15)	<0.001^*^
>60	-0.43 (-1.14, 0.28)	ref	ref	ref

**Table 4:** Mixed-effects regression models comparing length of stay across longer time frames The reference period is from March 31 to August 31, 2020. Longer time frames include two different time frames: the pre-COVID-19 lockdown (from March 31, 2017, to March 31, 2020) and the post-COVID-19 lockdown (from August 31, 2020, to August 31, 2022). ^β^Linear mixed-effects model for length of stay using a random intercept for month, robust standard errors, as well as age, gender, ethnicity, and appendicitis type as factors and covariates. ^*^Statistically significant result for alpha level < 0.05. "ref" served as the reference category for statistical comparisons; therefore, no p-value is reported for this group.

Time period	Unadjusted coefficients (95% CI)	p-value	Adjusted coefficients (95% CI)^β^	p-value
Intercept	1.51 (1.16-1.86)	<0.001^*^	1.43 (0.82, 2.04)	0.01^*^
March 31-August 31, 2020 (ref)	ref	ref	ref	ref
36 months preceding	0.31 (-0.02, 0.64)	0.07	0.36 (0.09, 0.63)	0.01^*^
24 months succeeding	0.19 (-0.22, 0.60)	0.37	0.24 (-0.12, 0.60)	0.18
Diagnosis type				
Simple appendicitis	1.25 (1.23, 1.27)	ref	ref	ref
Complicated appendicitis	2.22 (1.79, 2.65)	<0.001^*^	2.06 (1.75, 2.37)	<0.001^*^
Gender				
Male	1.90 (1.70, 2.10)	ref	ref	ref
Female	-0.20 (-0.40, 0.00)	0.05	0.01 (-0.13, 0.15)	0.9
Ethnicity				
Non-Hispanic or Latino	1.97 (1.54, 2.40)	ref	ref	ref
Hispanic or Latino	-0.17 (-0.68, 0.34)	0.53	-0.11 (-0.42, 0.20)	0.5
Age category				
0-10	-0.43 (-1.13, 0.27)	0.23	0.06 (-0.49, 0.61)	0.83
11-18	-1.27 (-1.77, -0.77)	<0.001^*^	-0.57 (-0.98, -0.16)	0.01^*^
19-40	-0.98 (-1.55, -0.41)	<0.001^*^	-0.47 (-0.90, -0.04)	0.03^*^
41-60	-1.18 (-1.73, -0.63)	<0.001^*^	-0.43 (-0.82, -0.04)	0.03^*^
>60	-0.43 (-1.13, -0.27)	ref	ref	ref

Adjusted odds for NOM, relative to the 2020 reference period, ranged from 0.56 to 1.46 across the years, with 2021 and 2022 showing higher odds (Table 5). Unadjusted and adjusted odds of NOM doubled in the 24 months following the COVID-19 lockdown compared to the preceding 36 months.

**Table 5:** Logistic regression models for non-operative management by time periods The reference period is from March 31 to August 31, 2020. Shorter time frames include five identical calendar periods from March 31 to August 31 for the years 2017, 2018, 2019, 2021, and 2022. Longer time frames include two different time frames: the pre-COVID-19 lockdown (from March 31, 2017, to March 31, 2020) and the post-COVID-19 lockdown (from August 31, 2020, to August 31, 2022). ^β^Adjusted for age, gender, ethnicity, and appendicitis type (simple and complicated). ^*^Statistically significant result for alpha level < 0.05. "ref" served as the reference category for all statistical comparisons; therefore, no p-value is reported for this group.

Time periods	Unadjusted odds ratio (95% CI)	p-value	Adjusted odds ratio (95% CI)^β^	p-value
2020 (ref)	ref	ref	ref	ref
Shorter time frames				
2017	1.04 (0.34-3.16)	0.95	1.33 (0.41-4.33)	0.63
2018	0.56 (0.16-1.96)	0.37	0.76 (0.21-2.78)	0.70
2019	0.69 (0.23-2.07)	0.51	0.67 (0.21-2.11)	0.49
2021	1.26 (0.49-3.24)	0.63	1.50 (0.56-4.02)	0.42
2022	1.46 (0.58-3.65)	0.42	1.42 (0.54-3.72)	0.48
Longer time frames				
36 months prior	0.98 (0.45-2.16)	0.96	1.13 (0.50-2.55)	0.69
24 months after	2.07 (0.96-4.46)	0.30	2.17 (0.96-4.69)	0.24

## Discussion

After analyzing the initial COVID-19 lockdown period in 2020, with various pre- and post-reference periods, we found no significant differences in the proportions of simple appendicitis, complicated appendicitis, or NOM across any of the comparator time frames. However, the duration of hospital stay was significantly reduced during the initial COVID-19 lockdown period.

Demographics

Patient demographics remained uniform across most time categories, with two exceptions during the 2021 and 2022 post-COVID-19 time frames. These periods showed slower mean heart rates and an increased reliance on CT scans for final diagnosis. The decreased heart rates were attributed to fewer children under 10 years of age being enrolled during these time frames. The heart rates of adults and adolescents are slower than those of young children [9]. Therefore, fewer children in these cohorts would decrease the mean heart rate. We believe two factors contributed to the lower pediatric ED volumes during this time: hospital renovations and the transition of the pediatric ED to an adult intensive care COVID-19 unit, with pediatric patients being managed in an adult ED environment. Regarding diagnostic methodology, we theorize that the increase in CT usage was secondary to extensive personal protection policies and efforts to limit contact with potentially COVID-19-positive patients, whereby ultrasound technicians were less inclined to perform detailed scanning, which resulted in more studies being inconclusive. Likewise, physicians may have opted for CT scans over US to expedite the ED course.

Primary outcomes

Our findings showed unchanged risks of complicated appendicitis, which contrasts with most published literature. A meta-analysis involving 54 studies from 22 countries reported a risk ratio for complicated appendicitis ranging from 1.32 in pediatric studies to 2.45 in mixed adult and pediatric populations [6]. Many authors suggested that this increase in complicated appendicitis cases was due to delayed access to medical care during the early pandemic, either from patient fear of contracting COVID-19 or public health policies that restricted ED use to severely ill patients [10-14]. We propose four reasons why our results differ from those of these studies.

First, our findings align with some well-powered publications that also reported unchanged risks of complicated appendicitis [15-22]. Well-powered studies are more reliable because they are less likely to produce false-negative results by minimizing the effect of bias and type I and II errors. This is supported by a meta-analysis from Andersson et al., which included 63 studies from 25 countries [23]. While the overall analysis of 100,059 patients showed a slightly increased incidence ratio (IR) of complicated appendicitis (IR 1.15; 95% CI 1.04-1.27), a sub-analysis of combined multicenter and regional data revealed that the incidence was, in fact, unchanged (IR 0.98; 95% CI 0.90-1.07). Therefore, despite being a relatively underpowered single-center study, our results mirrored the more robust findings from these larger, pooled data analyses.

Second, our results can be explained by the proportional variation of the data: the proportion of complicated appendicitis = absolute number of complicated cases/all cases of appendicitis (absolute numbers of complicated and simple appendicitis). Consequently, an increase in this proportion can result not only from an increase in complicated cases but also from a decrease in simple appendicitis cases. Multiple studies have demonstrated that during the COVID-19 lockdown, the absolute number of complicated cases remained relatively constant, while the total number of ED visits and simple appendicitis cases decreased. In the systematic review by Andersson et al. [23], they showed a decrease in the IR (0.66; 95% CI 0.59-0.73) of simple appendicitis, with single-center studies having the highest reduction (IR 0.62, 95% CI 0.53-0.73). This indicates that the observed increase in the risk of complicated appendicitis was likely driven by a reduction in simple cases, rather than a true rise in complicated ones. This is re-exemplified in a multicenter study in Germany, in which the COVID-19 lockdown in 2020 was compared to the same period in 2019 [24]. This analysis of 1,915 patients revealed an increased proportion of complicated appendicitis (64.4% versus 58.2%), despite a slight decrease in the absolute number of complicated appendicitis (597-569; p = 0.012). This drop in ED visits during the COVID-19 pandemic as reported in our study and other publications is reflective of successful public education efforts by healthcare officials on the importance of stay-at-home orders and reserving emergency departments for critically ill patients.

Third, our study design minimized the risk of over-classifying complicated appendicitis by using rigorous diagnostic criteria. The classification of simple versus complicated appendicitis was based solely on the earliest diagnostic modality performed upon arrival at the ED (ultrasonography or computed tomography). We excluded operative or pathological reports for initial diagnosis, because the time-sensitive clinical progression of acute appendicitis means that delays between initial radiological studies and surgery could artificially inflate the number of complicated cases. Furthermore, we avoided sole reliance on preset ICD-10 codes and instead conducted individual chart reviews to ensure a conclusive radiology diagnosis. The ambiguous terminology within the ICD-10 system often leads to the erroneous over-classification of complicated appendicitis [25,26]. For example, code K35.3 ("acute appendicitis with localized peritonitis") is typically classified as complicated appendicitis, even though subcodes K35.30 and K35.31 specify "absence of perforation," representing simple appendicitis. The accidental omission of these specific subcodes can lead to an incorrect classification.

Finally, our study's use of multiple pre- and post-pandemic time frames allowed us to account for baseline variability, which can confound results in studies using limited comparator periods. Many investigators have explored complication risks during the early pandemic using only one or two pre-pandemic reference points. Few studies have explored multiple earlier and later time frames [22,27]. For instance, our data revealed that the 2017 period, for unclear reasons, had significantly lower risks of simple appendicitis, and thus proportionally higher risks of complicated appendicitis, compared to subsequent years. This critical baseline observation would have been missed had the 2017 period been excluded as a reference category. Furthermore, the duration of the COVID-19 pandemic period analyzed varied significantly across different studies, ranging from a few weeks to several months, which may also contribute to the heterogeneity of published findings.

Secondary outcomes

The observed decrease in LOS during the early lockdown period is not surprising; it likely stemmed from institutional strategies to ensure resource availability for the anticipated surge of critically ill patients during the early phase of the pandemic. However, this local observation contrasts with a broader meta-analysis by Köhler et al., involving 23 studies, which showed no significant difference in mean LOS between COVID-19 and pre-pandemic periods [28].

The odds of receiving NOM were not substantially higher during the initial COVID-19 lockdown period; however, a considerable increase in the use of NOM occurred in the ensuing years. Emile et al. conducted a pooled analysis of 14 studies, including 2,140 patients, of whom 959 (44.8%) had NOM [29]. They reported that NOM during the pandemic was much more likely than before the COVID-19 pandemic (OR = 6.7, p < 0.001) [29]. In response to the challenges posed by the COVID-19 pandemic, the American College of Surgeons (ACS) and the Society of American Gastrointestinal and Endoscopic Surgeons (SAGES) published guidelines recommending a non-surgical approach (intravenous antibiotics followed by oral antibiotics upon clinical improvement) for the triage of general surgery patients [30]. The observed trend in NOM within our cohort may be explained by a delay in the adoption of the ACS and SAGES recommendations during the initial reference period, with surgeons subsequently incorporating the NOM approach during later waves of the pandemic.

Limitations

Several limitations warrant consideration. First, as a single-center retrospective study, our findings may have limited generalizability and are susceptible to selection bias, particularly regarding inter-facility transfers or miscoded diagnoses. Furthermore, we observed a potential diagnostic shift; increased CT utilization post-lockdown may have influenced the classification of appendicitis severity, introducing a degree of detection bias.

Second, our analysis was underpowered to detect moderate effect sizes. Specifically, the p-value of 0.07 for complicated appendicitis suggests the potential for a type II error, where a clinically significant difference may have been missed. While our statistical framework accounted for unequal time frames between study periods, residual confounding remains a possibility. Unmeasured factors, such as shifting patient thresholds for seeking care or evolving hospital triage protocols, may have influenced the results despite our adjustments for primary demographic and clinical variables. The power for simple versus complicated appendicitis compared between longer time frames was 55.80% at 5% level of significance. Similarly, it was only 15% for LOS. Given that the number of patients was 1,094, the magnitude of the differences should be evaluated critically along with the p-values.

Finally, the "initial lockdown" period does not coincide with the peak pandemic burden in Arizona (Winter 2021-2022). While this temporal heterogeneity is a factor, our primary objective was to evaluate the impact of the lockdown itself, and our 24-month follow-up period successfully captured subsequent viral peaks. Future multicenter research with larger cohorts is necessary to validate these trends across more granular pandemic phases.

## Conclusions

In this study, we found no statistically significant difference in the proportion of simple versus complicated appendicitis during the initial COVID-19 lockdown reference period compared to other time frames. Hospital LOS was shorter during the reference period and throughout 2021 when contrasted with other comparator periods, potentially indicating successful implementation of clinical pathways for efficient resource utilization. While proportions of NOM remained stable before and during the initial lockdown phase, NOM utilization increased in subsequent years, suggesting a shift in clinical practice.
